# Prognostic Value of Serum Cortisol, DHEA, and SOFA Score in Adult Sepsis Patients: A Retrospective Single-Center Clinical Study

**DOI:** 10.3390/jcm15155969

**Published:** 2026-07-30

**Authors:** Pinar Ayvat, Nurbanu Sezak, Günal Bilek, Ali Galip Ayvat, Mukaddes Colakoğulları, Gülşah Şehitoğlu Alpağut, Şeyda Kayhan Ömeroğlu, Hazal Koca

**Affiliations:** 1 Anesthesiology and Reanimation Department, Faculty of Medicine, Izmir Democracy University, 35140 Izmir, Turkey; drpinarunde@yahoo.com; 2 Infectious Diseases and Clinical Microbiology Department, Faculty of Medicine, Izmir Democracy University, 35140 Izmir, Turkey; nurbanu.sezak@idu.edu.tr; 3 Business Management Department, Faculty of Economics and Administrative Sciences, Izmir Democracy University, 35140 Izmir, Turkey; gunal.bilek@idu.edu.tr; 4 Project Management Department, Izmir Project Agency, 35210 Izmir, Turkey; 5 Biochemistry Department, Faculty of Medicine, Izmir Democracy University, 35140 Izmir, Turkey; m.colakogullari@idu.edu.tr; 6 Emergency Medicine Department, Izmir Buca Seyfi Demirsoy Education and Training Hospital, 35390 Izmir, Turkey; gulsahsehitoglu@gmail.com; 7 Anesthesiology Department, Izmir Dr. Suat Seren Chest Diseases and Chest Surgery Training and Research Hospital, 35170 Izmir, Turkey; seyda.kayhan@hotmail.com; 8 Anesthesiology and Reanimation Department, Izmir Buca Seyfi Demirsoy Education and Training Hospital, 35390 Izmir, Turkey; hazalkucukali67@gmail.com

**Keywords:** sepsis, SOFA score, dehydroepiandrosterone (DHEA), hydrocortisone, prognosis

## Abstract

**Background/Objectives:** Sepsis and septic shock remain leading causes of morbidity and mortality in intensive care units (ICUs) worldwide. While activation of the hypothalamic–pituitary–adrenal (HPA) axis is a vital adaptive response, the dissociation between cortisol and adrenal androgens like dehydroepiandrosterone (DHEA) is hypothesized to correlate with poor outcomes. This study aimed to evaluate the prognostic value of serum cortisol, DHEA, and the Cortisol/DHEA ratio in patients with sepsis admitted to the ICU. **Methods:** Clinical and laboratory data from 106 adult sepsis patients in a tertiary care hospital were retrospectively analyzed. Disease severity was assessed using Sequential Organ Failure Assessment (SOFA), Acute Physiology and Chronic Health Evaluation II (APACHE II), and Glasgow Coma Scale (GCS) scores. Baseline cortisol and DHEA levels were measured at the time of diagnosis. Logistic regression and Cox proportional hazards models were employed to identify predictors of in-hospital mortality and time to death, respectively. **Results:** A total of 106 adult sepsis patients with a mean age of 73.63 ± 13.70 years (55 female, 51.9%) were included in the study population. In the final logistic regression model, higher SOFA score was associated with greater odds of in-hospital mortality Odds ratio (OR) = 1.39 per 1-point increase, 95% Confidence interval (CI): 1.15–1.69, *p* < 0.001). Higher platelet count (PLT) was associated with lower odds of mortality (OR = 0.958 per 10 × 10^9^/L increase, 95% CI: 0.919–0.998, *p* = 0.042), whereas higher urea was associated with greater odds of mortality (OR = 1.112 per 10 mg/dL increase, 95% CI: 1.020–1.212, *p* = 0.016). The model area under the curve (AUC) was 0.7956. In univariable Cox analysis, higher baseline DHEA was associated with a greater hazard of death (Hazard ratio (HR) = 1.03, 95% CI: 1.014–1.046, *p* = 0.0002). In the final multivariable Cox proportional hazards model, baseline cortisol remained independently associated with the hazard of death (HR = 1.014, 95% CI: 1.007–1.022, *p* < 0.001), whereas DHEA was not retained. Notably, the Cortisol/DHEA ratio did not demonstrate independent prognostic value in either modeling approach. **Conclusions:** Higher baseline DHEA levels were associated with an increased hazard of death in univariable Cox proportional hazards analysis but did not retain independent prognostic significance in the final multivariable Cox proportional hazards model. While SOFA score remains the central prognostic tool, integrating continuous markers such as platelets and urea enhances risk stratification. The Cortisol/DHEA ratio offers no incremental prognostic utility over established clinical parameters.

## 1. Introduction

Sepsis is defined as life-threatening organ dysfunction caused by a dysregulated host response to infection [1]. Despite advances in critical care medicine, sepsis and septic shock remain major causes of morbidity and mortality in ICUs worldwide [2]. The pathophysiology of sepsis involves a complex interplay between pro-inflammatory and anti-inflammatory responses, in which the neuroendocrine system, particularly HPA axis, plays a central regulatory role [3].

Activation of the HPA axis during critical illness is considered a vital adaptive mechanism for survival, classically characterized by increased cortisol production. However, the prognostic value of serum cortisol levels alone remains a subject of debate due to factors such as alterations in corticosteroid-binding globulin and tissue resistance in critically ill patients [4,5]. The acute activation the HPA axis is an essential survival mechanism during critical illness, shifting metabolic resources and increasing circulating cortisol levels to maintain hemodynamic stability and regulate the immune response. In line with this, previous clinical studies have confirmed that serum cortisol concentrations are markedly elevated in patients with sepsis and septic shock compared to non-septic controls, reflecting the intensity of the systemic stress response [6]. Consequently, there is a need for novel biomarkers that can better reflect the functional status of the adrenal gland and the quality of the stress response.

DHEA and its sulfated form, dehydroepiandrosterone sulfate (DHEAS), are abundant adrenal steroids that serve as precursors to sex steroids but also possess distinct biological properties. While cortisol and adrenal androgens are secreted synchronously under physiological conditions, this relationship can be disrupted during severe illness. This phenomenon, described as “dissociation,” suggests a shift in adrenal steroidogenesis favoring cortisol production over DHEA and DHEAS during critical stress [7,8,9].

Experimental studies have demonstrated that DHEA possesses immunomodulatory properties, potentially acting as a functional antagonist to the immunosuppressive effects of cortisol and enhancing cellular immunity [10,11]. It has been hypothesized that a decrease in DHEA levels, or an increased Cortisol/DHEA ratio, may contribute to the state of “immunoparalysis” often observed in sepsis, thereby correlating with poor outcomes. Marx et al. [12] reported that DHEAS levels were preserved in survivors of severe sepsis but remained suppressed in nonsurvivors. Similarly, recent studies in community-acquired pneumonia and sepsis have indicated that the Cortisol/DHEA ratio may offer superior prognostic accuracy compared to cortisol alone or traditional scoring systems [13,14]. This acute endocrine disruption has also been documented in specific immunocompromised populations; for instance, a notable steroidogenic dissociation—characterized by elevated cortisol and deeply suppressed DHEAS levels—was independently associated with progressive inflammation, systemic hypoperfusion, and hospital mortality in cirrhotic patients presenting with septic shock [15].

The primary aim of this study is to investigate the prognostic value of serum cortisol and DHEA levels, as well as the Cortisol/DHEA ratio, in patients with sepsis admitted to the ICU. Furthermore, this study examines the complex relationships among these hormonal parameters, hematological metrics (including hemoglobin (HB) and PLTs), biochemical markers (such as urea, creatinine, and bilirubin), and critical care disease severity scores (SOFA). Identifying which of these multi-systemic parameters serves as the most decisive and independent predictor of patient mortality constitutes the secondary objective of this work.

## 2. Materials and Methods

### 2.1. Data Source and Study Population

This retrospective, single-center cohort study was conducted using clinical data collected from adult patients admitted to a tertiary care hospital. The exact study period spanned from March 2022 to March 2025. All participating individuals were managed within a specialized, tertiary-level (3rd-level) Anesthesiology ICU. Eligible participants were identified through a comprehensive screening of electronic medical records. 

To enter the study cohort, patients were required to meet the following inclusion criteria: (1) age ≥ 18 years, and (2) a formal diagnosis of sepsis or septic shock requiring active management within the ICU setting. Conversely, patients were excluded based on the following criteria: (1) age < 18 years, (2) failure to fulfill the official consensus definitions for sepsis or septic shock, (3) incomplete or missing primary clinical outcome data, or (4) active, systemic exogenous corticosteroid therapy prior to or during the baseline assessment window, which could confound endogenous hormone evaluation.

The operational criteria utilized to establish diagnoses were strictly aligned with the Third International Consensus Definitions for Sepsis and Septic Shock (Sepsis-3) guidelines. Sepsis was operationally defined as a documented or strongly suspected infection accompanied by an acute increase of ≥2 points in the SOFA score relative to the patient’s assumed baseline values. Septic shock was diagnosed in patients who met the criteria for sepsis but exhibited persistent hypotension requiring vasopressor support to maintain a mean arterial pressure ≥ 65 mmHg and a serum lactate concentration > 2 mmol/L despite adequate volume fluid resuscitation.

The exact time point defined as “baseline” was restricted to the clinical, demographic, and laboratory parameters obtained within the first 24 h of ICU admission and initial sepsis diagnosis. Laboratory measurements obtained during this initial 24 h baseline window were denoted with the suffix “1” (e.g., Cortisol1), while corresponding follow-up measurements obtained on day 3 of the study were denoted with the suffix “2” (e.g., Cortisol2) to assess longitudinal profile trends.

For the time-to-event survival analysis, the time origin was fixed at the exact date of sepsis onset. The standardized follow-up duration was set to 28 days. Patients were tracked continuously through their ICU stay, during eventual transfers to step-down wards, or until death occurred. To handle hospital discharge or step-down unit transfers accurately within the survival design, patients discharged alive from the ICU before the completion of the follow-up period were actively monitored up to day 28 to ascertain their definitive survival status. Standard right-censoring rules were applied at day 28 for all individuals who survived beyond this standardized observation window.

The study was conducted in accordance with the principles of the Declaration of Helsinki, and the protocol was formally reviewed and approved by the local institutional ethics committee. Given the observational, retrospective nature of the data collection from routine medical registries, the requirement for obtaining written informed consent was waived by the institutional board. The anonymized raw clinical data, severity scores, and laboratory metrics analyzed during this study are provided in Supplementary File S1.

### 2.2. Variables and Outcomes

The study variables comprised demographic characteristics, clinical severity scores, and laboratory parameters. Demographic data included age and sex. Clinical severity was assessed using SOFA score, APACHE II score, and GCS. Laboratory variables obtained at baseline included complete blood count parameters [white blood cell count (WBC), neutrophil percentage (NEU), PLT, and HB], renal function markers [serum creatinine, urea, and estimated glomerular filtration rate (eGFR)], inflammatory and infection-related biomarkers [C-reactive protein (CRP), procalcitonin (PCT), and lactate], liver function parameters (bilirubin, albumin), and endocrine stress markers (cortisol and DHEA). When available, repeated measurements of selected laboratory parameters were recorded to assess internal consistency. The primary outcome was in-hospital mortality, recorded as a binary variable (survivor vs. non-survivor). A secondary outcome was time to death after sepsis onset, which was analyzed using Cox proportional hazards regression.

### 2.3. Measurements

Venous blood samples for endocrine parameters were collected from all participants within the first 24 h of their sepsis diagnosis. To minimize the confounding effect of the physiological diurnal rhythm on adrenal steroidogenesis, blood sampling was strictly standardized to a morning window between 06:00 AM and 08:00 AM. Serum cortisol and DHEA levels were quantified using standard Enzyme-Linked Immunosorbent Assay (ELISA) and Electrochemoluminescence Immunoassays (ECLIA) on automated laboratory platforms. As noted in our selection criteria, none of the included patients had received exogenous systemic corticosteroid therapy prior to or during the baseline sample collection window.

### 2.4. Statistical Analysis

Continuous variables were summarized as mean ± standard deviation and compared between survivors and non-survivors using Welch’s *t*-test. Categorical variables were summarized as *n* (%) and compared using Fisher’s exact test. Missing values were excluded on a variable-by-variable basis for descriptive analyses and by complete-case analysis for multivariable models.

Receiver operating characteristic (ROC) curve analyses were performed to evaluate the discriminative performance of SOFA, APACHE II, and GCS scores for in-hospital mortality. AUC values were calculated with 95% confidence intervals where appropriate, and optimal cut-off values were identified using the Youden index.

Correlation analyses were performed using Pearson’s correlation coefficients to evaluate linear relationships among continuous variables, including endocrine biomarkers, disease severity scores, and laboratory parameters. Correlation analysis was primarily conducted to describe pairwise associations among candidate predictors. In addition, multicollinearity was formally assessed using variance inflation factors (VIFs) and tolerance statistics. VIF values <5 and tolerance values >0.20 were considered indicative of the absence of problematic multicollinearity.

To evaluate factors associated with in-hospital mortality, univariable logistic regression analyses were initially conducted. Variables demonstrating a statistically significant association (*p* < 0.05) or borderline significance (*p* ≤ 0.10) were considered for multivariable logistic regression. To reduce redundancy and limit model complexity, follow-up laboratory measurements and conceptually overlapping severity scores were excluded a priori from the multivariable candidate set. Six baseline candidate predictors were therefore considered for the final multivariable logistic regression model, corresponding to approximately 12.3 deaths per candidate predictor based on 74 in-hospital deaths. Backward elimination was then used to obtain a final multivariable logistic regression model, with statistical significance, clinical interpretability, multicollinearity diagnostics, and changes in model discrimination considered jointly. The potential instability and bias associated with data-driven variable selection were addressed through bootstrap internal validation and are acknowledged as a study limitation.

Time-to-event analyses were performed using Cox proportional hazards regression to estimate associations with the hazard of death while accounting for follow-up time and censoring. Variables showing a statistically significant association (*p* < 0.05) or borderline significance (*p* ≤ 0.10) in univariable Cox analyses were considered for multivariable modeling. After excluding follow-up measurements and conceptually redundant severity scores, 10 baseline candidate predictors were considered. The complete-case final Cox dataset contained 71 deaths, corresponding to approximately 7.1 deaths per initial baseline candidate predictor as a descriptive reference. Backward elimination was applied with attention to clinical interpretability and Harrell’s concordance index (C-index). The proportional hazards assumption was evaluated using Schoenfeld residuals, and no significant global violation was detected.

Internal validation was performed using 1000 bootstrap resamples to estimate optimism-corrected model performance. For the final multivariable logistic regression model, optimism-corrected AUC and Brier score were calculated. For the Cox proportional hazards model, optimism-corrected Harrell’s C-index was estimated.

Apparent calibration of the final multivariable logistic regression model was assessed in the same cohort used for model development. Patients were divided into 10 groups (deciles) according to their predicted mortality probabilities, and the mean predicted probability was compared with the observed mortality rate within each group. A locally estimated scatterplot smoothing (LOESS) curve was overlaid to visualize agreement between predicted and observed mortality probabilities across the risk range. The 45-degree diagonal line represented perfect calibration. Because calibration was assessed in the development cohort, it was interpreted as apparent calibration rather than independently validated calibration. Internal validation was additionally performed using 1000 bootstrap resamples, with optimism-corrected estimates of discrimination and calibration and Brier scores used to assess overall prediction error.

All statistical tests were two-sided, and a *p*-value < 0.05 was considered statistically significant unless otherwise specified. Analyses were performed using R version 4.5.0, with appropriate packages for regression modeling, survival analysis, and model validation.

## 3. Results

### 3.1. Characteristics of the Study Population

Baseline clinical, laboratory, and endocrine characteristics stratified by survival status are presented in Table 1. Among the 106 patients included in the study, 32 survived and 74 died during hospitalization. Non-survivors were older than survivors, although this difference did not reach statistical significance. Disease severity was higher among non-survivors, as reflected by significantly higher SOFA scores and borderline higher APACHE II scores. Non-survivors also had significantly lower HB, PLT, and albumin levels, while urea and lactate levels were significantly higher. Regarding endocrine biomarkers, baseline cortisol and DHEA levels were significantly higher among non-survivors, whereas the cortisol/DHEA ratio did not differ significantly between survival groups.

### 3.2. Correlation Analysis

Correlation analyses were performed to examine the relationships among the study variables. In particular, age, disease severity scores (including SOFA and APACHE II), and biochemical biomarkers were jointly evaluated to better understand their interdependencies and to assess the suitability of these variables for inclusion in subsequent multivariable analyses.

**Figure 1:**
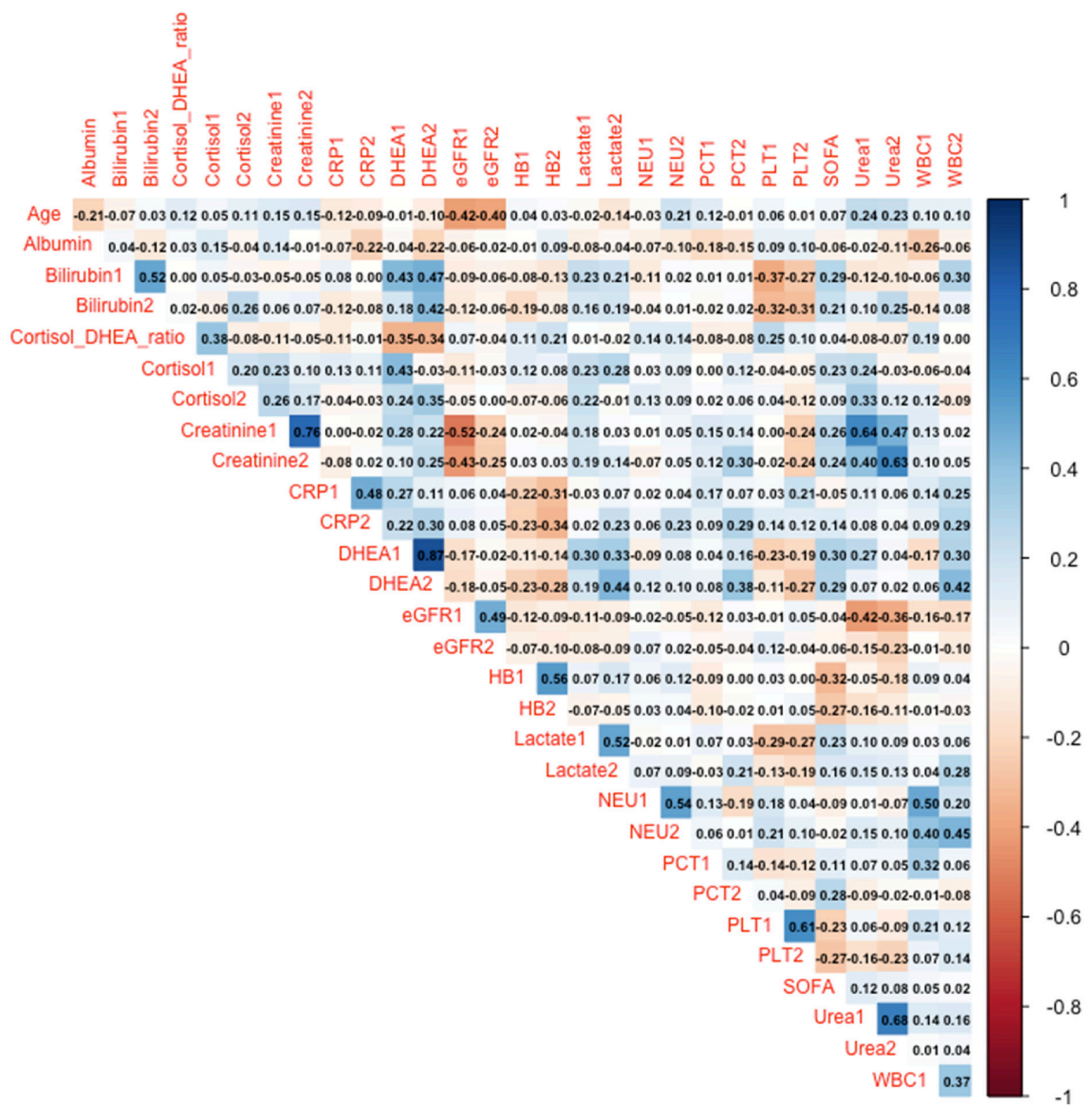
Correlation matrix of clinical severity scores, laboratory parameters, and endocrine biomarkers, including cortisol, DHEA, and the Cortisol/DHEA ratio.

Figure 1 illustrates the correlation matrix and corresponding correlation plot depicting pairwise associations among age, clinical scores, and laboratory variables. The visualization provides an integrated overview of correlations between patient demographics, severity of illness indicators, inflammatory markers, metabolic parameters, and hormonal measurements. The strength and direction of correlations are represented by the size and color intensity of the coefficients, facilitating the identification of meaningful patterns and potential collinearity.

Correlation analysis revealed predominantly weak to moderate associations among the studied clinical, laboratory, and endocrine variables. Strong positive correlations were mainly observed between repeated measurements of the same biomarkers, indicating internal consistency of the laboratory data. In particular, creatinine, urea, lactate, PLT, and DHEA levels measured at different time points showed positive correlations with their corresponding repeated measurements.

Moderate positive correlations were identified among selected inflammatory and hematological markers. CRP showed positive associations with PCT and WBC-related parameters, reflecting the expected inflammatory response pattern in critically ill septic patients. Negative associations were observed between HB and inflammatory markers, as well as between PLT and selected markers of organ dysfunction, supporting the relationship between systemic inflammation, hematological impairment, and disease severity.

Regarding endocrine biomarkers, baseline cortisol showed weak to moderate positive correlations with SOFA score, urea, creatinine, and lactate. Baseline DHEA showed moderate positive correlations with SOFA score, bilirubin, creatinine, CRP, and lactate, while showing a negative association with PLT.

The cortisol/DHEA ratio was also included in the correlation matrix to evaluate whether this combined endocrine stress index demonstrated stronger associations with disease severity or inflammatory markers than the individual hormone measurements. The ratio showed only weak correlations with SOFA score, CRP, PCT, lactate, urea, creatinine, and albumin, while it was moderately positively correlated with cortisol and moderately negatively correlated with DHEA.

Importantly, formal assessment of multicollinearity confirmed the absence of problematic collinearity among candidate predictors. VIFs were uniformly low (all VIFs < 2.0), and tolerance values exceeded 0.75 for all variables, supporting the inclusion of these predictors in subsequent multivariable regression analyses.

### 3.3. Logistic Regression and Mortality Prediction

**Figure 2:**
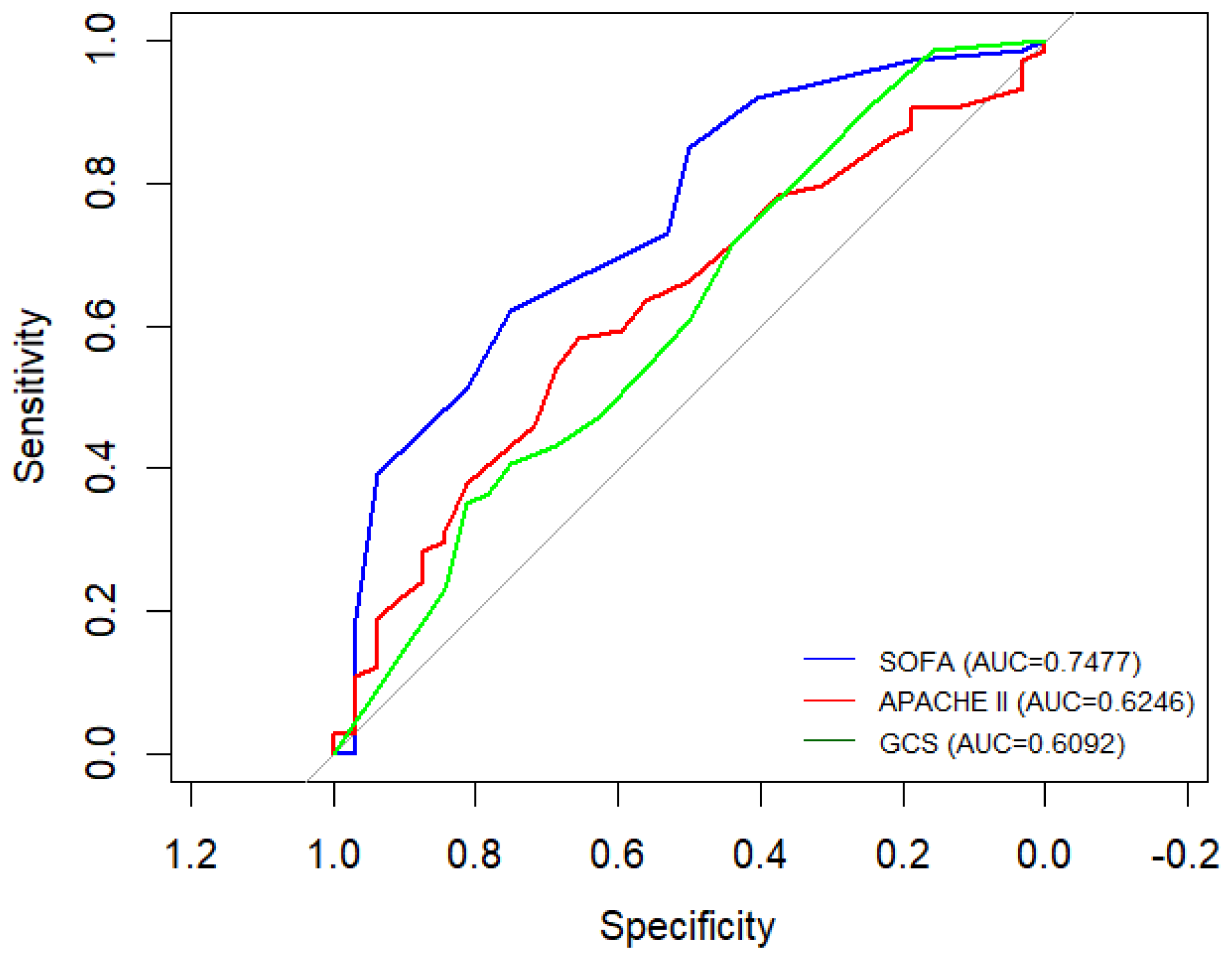
ROC curves for SOFA, APACHE II, and GCS scores in predicting in-hospital mortality. The gray diagonal line represents the no-discrimination reference line (AUC = 0.50).

Accordingly, univariate logistic regression analyses were conducted to assess the prognostic relevance of clinical severity scores for in-hospital mortality and to guide subsequent multivariable model development. In univariate logistic regression analysis, SOFA score was significantly associated with mortality (OR = 1.41 per point increase, *p* = 0.0002). In contrast, APACHE II and GCS showed only borderline associations with mortality (*p* = 0.068 and *p* = 0.078, respectively). Figure 2 presents the ROC curves of SOFA, APACHE II, and GCS scores for predicting in-hospital mortality among patients with sepsis. As illustrated in Figure 2, SOFA score demonstrated the highest discriminative performance, with an AUC of 0.75, indicating acceptable predictive accuracy.

Using the Youden index, the optimal SOFA cut-off value for mortality prediction was identified as 9.5, corresponding clinically to a SOFA score ≥10. At this threshold, SOFA score achieved a sensitivity of 62% and a specificity of 75%. This indicates that a SOFA score of 10 or higher correctly identified approximately 62% of patients who died, while correctly excluding 75% of survivors. However, the sensitivity of 62% indicates that 38% of patients who died were not identified by this threshold; therefore, the SOFA cut-off should be interpreted as a supportive risk-stratification measure rather than a standalone clinical decision rule.

To further improve the predictive performance beyond that achieved by SOFA score, multivariable logistic regression modeling was subsequently employed. Candidate variables for the multivariable logistic regression model were selected based on univariate logistic regression analyses. Only variables showing a statistically significant association with mortality (*p* < 0.05) or borderline significance (*p* ≤ 0.10) were considered eligible for further evaluation. Based on univariate analyses, the following numerical variables met the predefined inclusion criteria: SOFA score (*p* < 0.001), Urea2 (*p* = 0.002), PLT2 (*p* = 0.005), HB1 (*p* = 0.020), PLT1 (*p* = 0.025), Urea1 (*p* = 0.027), Cortisol/DHEA (*p* = 0.031), APACHE II score (*p* = 0.069), HB2 (*p* = 0.072), GCS (*p* = 0.078), Lactate1 (*p* = 0.081), Albumin (*p* = 0.083), Lactate 2 (*p* = 0.101), and age (*p* = 0.104).

To ensure clinical interpretability and model stability, follow-up (second) laboratory measurements were not included in the multivariable model. Although some second measurements (e.g., Urea2, PLT2) demonstrated significant associations in univariate analyses, these variables are highly correlated with their baseline counterparts and primarily reflect disease progression rather than baseline risk. Inclusion of both time points could introduce redundancy and collinearity; therefore, baseline measurements were preferentially selected. Similarly, among clinical severity scores, only SOFA score was included in multivariable modeling. Other severity indices (e.g., APACHE II and GCS), despite borderline significance in univariate analyses, were excluded due to conceptual overlap with SOFA and their limited additional contribution to model discrimination.

A stepwise backward elimination approach was then applied. At each step, the variable with the largest non-significant *p*-value was removed, and model performance was reassessed. Model discrimination was evaluated using the area under ROC curve (AUC). Variables that were not statistically significant were excluded because their inclusion did not result in a meaningful improvement in AUC, nor did their removal lead to a substantial decrease in predictive performance. Consequently, only variables with statistically significant contributions were retained in the final model to achieve a parsimonious and clinically robust predictive model. Accordingly, the final multivariable model retained only SOFA, PLT, and Urea, achieving a balance between parsimony and predictive accuracy while remaining firmly grounded in both univariable screening results and overall model performance. Although PLT is included as a component of SOFA score, it is incorporated in a categorical, threshold-based format, whereas in our model, PLT was included as a continuous variable. This allows platelet levels to provide additional prognostic information beyond the ordinal SOFA sub-score. Furthermore, no evidence of problematic multicollinearity was observed between SOFA score and PLT because the correlation between them is −0.23 (Figure 1). Importantly, exclusion of PLT led to a decrease in model discrimination (AUC), supporting its independent and incremental prognostic value.

The model is given in Table 2. Accordingly, SOFA score was the strongest prognostic factor in the model. Each one-point increase in SOFA score was associated with a 39% increase in the odds of mortality (OR = 1.39, *p* < 0.001), highlighting the critical impact of progressive multiorgan dysfunction on patient outcomes.

Bootstrap internal validation supported the internal performance of the final multivariable logistic regression model and indicated limited optimism. The apparent AUC was 0.7956, with a mean optimism of 0.0203, resulting in an optimism-corrected AUC of 0.7753 after 1000 bootstrap resamples. The apparent Brier score was 0.1622, and the bootstrap mean Brier score was 0.1692. Apparent calibration yielded an intercept of 0.000 and a slope of 1.000. After bootstrap correction, the optimism-corrected calibration intercept was 0.001 and the optimism-corrected calibration slope was 0.901, suggesting limited overfitting and no substantial systematic miscalibration within the development cohort.

In addition to global organ dysfunction, alterations in hematological parameters were also independently associated with mortality. PLT was inversely associated with mortality. Higher platelet levels were independently linked to a reduced risk of death (OR = 0.996 per unit increase, *p* = 0.042). In contrast, the urea level was positively associated with mortality risk. Each 1 mg/dL increase in urea corresponded to an approximately 1% increase in the odds of death (OR = 1.01, *p* = 0.016).

Importantly, the model demonstrated acceptable discriminative performance (AUC = 0.7956), indicating that such individual risk estimates were generated within a framework capable of reliably distinguishing survivors from non-survivors. Overall, this parsimonious model integrates markers of multiorgan failure, hematological status, and renal function, providing a clinically coherent framework for mortality risk stratification in patients with sepsis.

To illustrate the clinical applicability of the final multivariable logistic regression model, a patient with a SOFA score of 15, PLT of 300 × 10^9^/L, and urea level of 50 mg/dL has an estimated mortality probability of 88.6%, as shown below.

$$P\left(Mortality\right)=\frac{1}{1+e^{-(-2.135+0.330*Sofa+0.0106*Ure-0.0043*Platelet)}}=\frac{1}{1+e^{-(-2.135+0.330*15+0.0106*50-0.0043*300)}}=0.886$$

**Figure 3:**
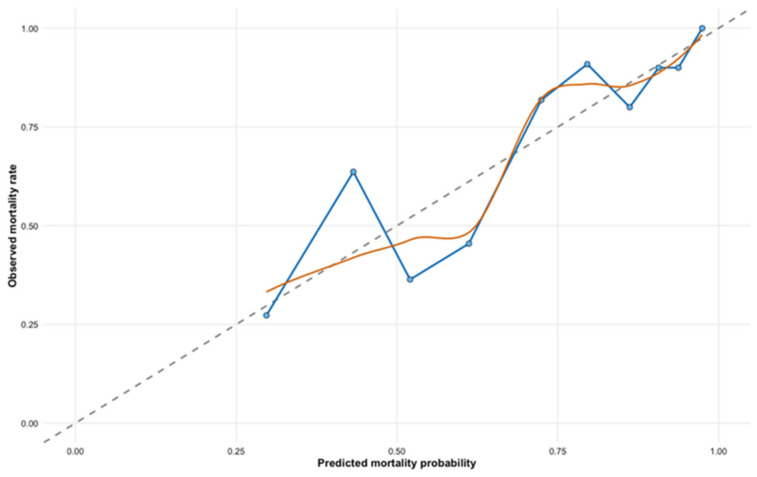
Apparent calibration plot of the final multivariable logistic regression model for in-hospital mortality. The blue line and points represent the observed mortality rates across the risk groups, the orange line represents the LOESS curve, and the gray dashed diagonal line represents perfect calibration.

Apparent calibration of the final multivariable logistic regression model was evaluated by comparing predicted and observed mortality probabilities across 10 risk groups and by overlaying a LOESS curve (Figure 3). The apparent calibration intercept was 0.000 and the apparent calibration slope was 1.000. After 1000 bootstrap resamples, the optimism-corrected calibration intercept was 0.001 and the optimism-corrected calibration slope was 0.901, indicating limited overfitting and no substantial systematic miscalibration within the development cohort. The apparent Brier score was 0.1622, while the bootstrap mean Brier score was 0.1692. Because calibration was assessed in the same cohort used for model development, these findings should be interpreted as evidence of internal model performance rather than independent validation of clinical reliability or generalizability.

### 3.4. Cox Proportional Hazards Analysis

To complement the binary mortality analyses and to account for differences in the timing of death after sepsis onset, time-to-event analyses were performed using Cox proportional hazards regression. In univariable Cox analyses, several clinical and laboratory parameters were associated with the hazard of death. Candidate variables for the multivariable Cox proportional hazards model were selected based on the results of univariable Cox regression analyses. Variables showing a statistically significant association with mortality (*p* < 0.05) or borderline significance (*p* ≤ 0.10) were considered eligible for further evaluation.

Based on univariate Cox analyses, the following variables met the predefined selection criteria: SOFA score (*p* < 0.001), Lactate1 (*p* < 0.001), Lactate2 (*p* < 0.001), Cortisol1 (*p* < 0.001), DHEA1 (*p* < 0.001), DHEA2 (*p* < 0.001), PLT1 (*p* < 0.001), PLT2 (*p* = 0.001), Creatinine1 (*p* = 0.09), Creatinine2 (*p* = 0.012), Albumin (*p* = 0.013), Bilirubin1 (*p* = 0.017), Bilirubin2 (*p* = 0.036), Urea1 (*p* = 0.059), and age (*p* = 0.078).

To ensure clinical interpretability and model stability, follow-up (second) laboratory measurements were not included in the final multivariable Cox proportional hazards model, despite their statistical significance in univariate analyses. These measurements primarily reflect disease progression over time and are highly correlated with baseline values. Including both time points could introduce redundancy and collinearity; therefore, baseline measurements were preferentially selected. Similarly, among clinical severity indices, only SOFA score was included in multivariable survival modeling. Other scores were excluded due to conceptual overlap with SOFA and their limited additional prognostic contribution.

A stepwise backward elimination approach was applied during multivariable Cox model construction. At each step, variables with the largest non-significant *p*-values were sequentially removed, and model performance was simultaneously evaluated using Harrell’s C-index. Non-significant variables were excluded when their inclusion did not lead to a meaningful improvement in concordance. This strategy allowed the construction of a parsimonious and clinically interpretable survival model while preserving discriminatory performance.

The final multivariable Cox proportional hazards model is given in Table 3. Accordingly, SOFA score, Lactate1, Cortisol1, Albumin1, and Platelet1 count remained independently associated with mortality. The model demonstrated good discriminatory ability, with a C-index of 0.744, indicating acceptable survival prediction performance. Overall, the model showed strong global significance, as indicated by the likelihood ratio (LR), Wald, and log-rank tests (all *p* < 0.001), confirming that the selected variables collectively provide meaningful prognostic information for mortality risk. Also, the proportional hazards assumption was formally assessed using Schoenfeld residuals. No significant violations of the proportional hazards assumption were detected at the global level, supporting the validity of the Cox model and the interpretation of HRs as constant over time.

Across the final multivariable models, SOFA score and PLT were retained in both the final multivariable logistic regression model and Cox proportional hazards models. Urea was retained only in the final multivariable logistic regression model, whereas cortisol, lactate, and albumin were retained only in the final multivariable Cox proportional hazards model. Baseline DHEA was significantly associated with the hazard of death in univariable Cox analysis but was not retained in the final multivariable Cox proportional hazards model. The cortisol/DHEA ratio was significantly associated with in-hospital mortality in univariable logistic regression analysis (*p* = 0.031), but this association did not persist after multivariable adjustment (*p* = 0.583); the ratio was also not significantly associated with the hazard of death in univariable Cox analysis (HR = 1.04, 95% CI: 0.97–1.11, *p* = 0.291).

## 4. Discussion

Logistic regression and Cox proportional hazards regression provided complementary information regarding prognosis in this cohort. Logistic regression modeled in-hospital mortality as a binary outcome, whereas Cox regression estimated associations with the hazard of death while accounting for follow-up time and censoring. Across both final multivariable models, SOFA score and PLT showed consistent prognostic associations. Higher SOFA scores were associated with both greater odds of in-hospital mortality and a higher hazard of death, reinforcing the central prognostic role of organ dysfunction severity in sepsis. Similarly, higher PLTs were associated with lower odds of in-hospital mortality and a lower hazard of death, supporting the prognostic relevance of thrombocytopenia in critically ill patients.

Differences between the two final models were also observed. Urea was independently associated with in-hospital mortality in the final multivariable logistic regression model but was not retained in the final multivariable Cox proportional hazards model. In contrast, cortisol, lactate, and albumin were not retained in the final multivariable logistic regression model but remained independently associated with the hazard of death in the final multivariable Cox proportional hazards model. These differences likely reflect the distinct outcome structures addressed by the two approaches rather than contradictory findings, because logistic regression evaluates a binary endpoint whereas Cox regression incorporates follow-up time and censoring.

Regarding the endocrine biomarkers that constituted the primary focus of the study, baseline DHEA was significantly associated with the hazard of death in univariable Cox analysis but was not retained in the final multivariable Cox proportional hazards model. Accordingly, this association should be interpreted as unadjusted rather than independent. The cortisol/DHEA ratio showed a significant association with in-hospital mortality in univariable logistic regression analysis (*p* = 0.031), but this association was attenuated after multivariable adjustment (*p* = 0.583). The ratio was also not significantly associated with the hazard of death in univariable Cox analysis (HR = 1.04, 95% CI: 0.97–1.11, *p* = 0.291). Collectively, these findings suggest that although individual endocrine biomarkers showed associations with prognosis, the cortisol/DHEA ratio did not provide independent prognostic information beyond established clinical and laboratory predictors.

Despite the availability of multiple risk scoring systems for monitoring high-risk patients in intensive care, none of them are sufficiently accurate to predict mortality with adequate sensitivity and specificity [16]. The inclusion of new biomarkers represents a fundamental task that can improve prognostic capability and guide therapeutic interventions in septic patients. The dramatic alterations in cortisol and DHEA levels observed during severe sepsis reflect a well-documented ‘adrenal steroidogenic shift’ or ‘cortisol–androgen dissociation.’ Under conditions of hyperinflammation and septic shock, sustained activation of the HPA axis, driven by pro-inflammatory cytokines, shifts the enzymatic pathways within the adrenal cortex [6].

Activation of the HPA axis is a key component of the overall response to illness [5]. During sepsis, upregulation of adrenal androgens such as cortisol and DHEA has been described, but this has not been observed for DHEAS. In one cross-sectional study involving 181 patients with septic shock, 31 patients with acute trauma, and 60 healthy controls, serum cortisol, DHEA, and DHEAS were measured before and 60 min after adrenocorticotropic hormone (ACTH) stimulation [8]. In both septic shock and trauma patients, serum cortisol was elevated and DHEAS was decreased (*p* < 0.001), whereas DHEA was significantly increased in sepsis compared to healthy controls (*p* < 0.001); notably, neither cortisol nor DHEA showed a significant further rise after ACTH stimulation in sepsis, and most critically ill patients exhibited higher cortisol (*p* = 0.069), lower DHEA (*p* = 0.076), and significantly higher cortisol/DHEA ratios (*p* = 0.004); similarly, non-survivors of septic shock had markedly elevated cortisol/DHEA ratios, while survivors showed ratios comparable to controls.

Numerous studies have demonstrated the role of cortisol and adrenal androgens as prognostic factors in septic patients [3,12,14,17,18]. For example, in a study of 179 cases where disease severity was assessed using the Pneumonia Severity Index, Kaplan–Meier analysis revealed a higher risk of death in patients in the highest deciles of cortisol, DHEA, and cortisol/DHEAS ratio. The authors indicated that cortisol, DHEAS, and their ratios are associated with the severity of community-acquired pneumonia, and that cortisol and DHEA can be used to predict mortality. Adrenal function may be an important factor in the outcomes of severe pneumonia [14]. A dissociation between cortisol and DHEAS, characterized by elevated cortisol and reduced DHEAS levels, was observed in cirrhotic patients also who died from septic shock. A lower DHEAS-to-cortisol ratio was associated with greater disease severity, increased inflammation, and CIRCI, suggesting its potential value as a prognostic marker [15].

Considering their immunomodulatory effects, it has been proposed that increases in the cortisol/DHEA and cortisol/DHEAS ratios could represent novel prognostic markers in septic patients [8,12,14]. A previous study investigated the time-dependent changes in DHEA, DHEAS, cortisol, adrenocorticotropin, and inflammatory variables in survivors (*n* = 15) and non-survivors (*n* = 15) of severe sepsis. During early sepsis, DHEAS levels (measured as a percentage of age-matched normal levels) were significantly higher in survivors than in non-survivors. DHEAS levels decreased in survivors over time (p=0.0001) but remained consistently low in non-survivors during late sepsis. Similarly, DHEA levels did not show a significant rise in survivors compared to non-survivors during early sepsis. DHEA subsequently decreased in survivors during late sepsis (p<0.01), whereas no such decrease was observed in the non-survivor group. Ultimately, the DHEAS/cortisol ratio decreased significantly in both survivors and non-survivors, while the DHEA/cortisol ratio decreased exclusively in survivors [12].

The pattern of adrenal–androgen dissociation is also highly consistent with specialized, high-risk critical care models, such as cirrhotic patients who develop spontaneous bacterial peritonitis. In the presence of localized peritoneal infections and severe systemic inflammation, non-surviving patients similarly exhibit a profound reduction in the DHEAS to cortisol ratio. This lower ratio correlates directly with acute pro-inflammatory cytokine surges, elevated baseline SOFA scores, and increased hospital mortality, further establishing the cortisol–androgen imbalance as a universal surrogate for critical illness severity [19].

This inflammation-driven neuroendocrine dissociation is not unique to classic bacterial sepsis; it represents a unified pathological hallmark across severe viral hyperinflammatory states as well, most notably demonstrated in COVID-19. Clinical investigations mapping the adrenal profile of hospitalized COVID-19 patients have revealed an identical pattern of severe steroidogenic disruption. Specifically, severe and critically ill viral patients demonstrate significantly elevated baseline cortisol concentrations alongside a marked depletion of adrenal androgens like DHEAS, resulting in a profoundly elevated cortisol/DHEAS ratio compared to mild cases and healthy controls. Crucially, this specialized neuroendocrine shift demonstrates an intricate biological link to systemic hyperinflammation; an increased cortisol/adrenal androgen ratio correlates directly with acute phase reactants and cellular damage markers [9]. These cumulative findings from viral sepsis models reinforce our own observations in general ICU sepsis.

According to our findings, although baseline cortisol and DHEA levels were significantly higher among non-survivors, the cortisol/DHEA ratio did not show a significant association with either time to death (HR = 1.04, 95% CI: 0.97–1.11, *p* = 0.291) or in-hospital mortality (OR ≈ 1.04, *p* = 0.583). Furthermore, this ratio failed to achieve independent statistical significance in the final multivariable Cox proportional hazards model and logistic regression models that included established prognostic factors such as SOFA score. Although a previous study reported a higher cortisol/DHEA ratio in non-survivors of septic shock, whether this ratio is a clear prognostic tool independent of clinical scores remains debatable [8]. Indeed, our findings parallel previous research indicating that the cortisol/DHEA ratio does not provide a superior predictive ability over cortisol alone for predicting short- and medium-term (in-hospital or 28-day) mortality, and that adrenal ratios may only become significant in long-term (90-day) follow-ups [13]. During the acute phase of sepsis, since parameters such as baseline DHEA and SOFA score [16]—which reflects the degree of rapid tissue-level metabolic breakdown and organ dysfunction—provide sufficient prognostic information on their own, calculating the ratio of these two hormones may not offer an additional independent contribution in multivariable models.

Several factors may explain why the cortisol/DHEA ratio failed to demonstrate independent prognostic value in our study, despite contrary findings in the literature. First, the overriding statistical weight of acute organ dysfunction metrics—specifically the SOFA score, PLT, and serum urea—entirely attenuated the ratio’s predictive power in our multivariable models, suggesting it functions primarily as a collinear surrogate of global tissue-level injury rather than an independent prognostic driver. Second, our cohort’s advanced mean age (73.63 ± 13.70 years) may have narrowed the variance of this endocrine metric, as adrenal androgen synthesis naturally and severely declines with age. Finally, while acute individual hormone elevations capture immediate survival trajectories, complex composite endocrine ratios may require longer follow-up windows (e.g., 90 days) to reveal their impact on persistent immunoparalysis, rendering them less sensitive for predicting short-term in-hospital mortality.

In our cohort, SOFA score remained the dominant predictor of mortality in multivariable analysis, consistent with the conceptual purpose of SOFA as a structured summary of acute multiorgan dysfunction. Mortality after sepsis onset was evaluated using both logistic regression and Cox proportional hazards models in order to capture complementary aspects of outcome prediction. While logistic regression was used to identify factors associated with the occurrence of mortality, Cox regression enabled assessment of the timing of death, thereby providing a more nuanced understanding of sepsis-related outcomes.

Across both modeling approaches, SOFA score emerged as the most consistent and robust predictor. In logistic regression, higher SOFA scores were independently associated with increased odds of mortality, reflecting the established role of cumulative organ dysfunction in determining overall survival. This finding was reinforced by Cox regression analyses, in which higher SOFA scores were associated with an increased hazard of death, indicating that greater organ failure burden not only increased the likelihood of mortality but also accelerated its occurrence. The concordance of SOFA across both models underscores its central role in sepsis prognostication and is consistent with other studies in the literature [20,21,22]. Recent syntheses and comparative studies generally report that SOFA outperforms simpler screening tools such as Systemic inflammatory response syndrome (SIRS), National Early Warning Score (NEWS) and often performs similarly or better than alternative scoring approaches [23,24].

Beyond SOFA, we identified PLT and urea as independent predictors, which is clinically plausible and consistent with sepsis pathobiology. Thrombocytopenia is commonly interpreted as a marker of sepsis-associated coagulopathy, endothelial activation, and microvascular injury. Similarly, elevated urea likely reflects renal dysfunction, systemic hypoperfusion, and catabolic stress. Importantly, these parameters provided incremental prognostic information beyond the aggregated SOFA score, suggesting that continuous laboratory measures may capture risk gradients not fully represented by SOFA’s categorical component thresholds.

PLT demonstrated a protective association in both the final multivariable logistic regression model and the final multivariable Cox proportional hazards model, suggesting that thrombocytopenia is associated with greater in-hospital mortality risk and a higher hazard of death during follow-up. These findings support the prognostic relevance of PLT in sepsis and are consistent with previous reports linking thrombocytopenia to disease severity, coagulopathy, and adverse outcomes [25,26].

Baseline urea level showed a similar pattern. Its association with mortality in the final multivariable logistic regression model and in the final multivariable Cox proportional hazards model indicates that renal dysfunction contributes to both the risk and timing of death in sepsis. However, urea did not remain significant in the Cox models, suggesting that its prognostic effect may overlap with other markers of systemic illness severity rather than representing an independent driver of early mortality [27].

In contrast, lactate level emerged as a strong and consistent predictor in Cox regression analyses but was not emphasized in the final multivariable logistic regression model. This finding highlights an important distinction between the two modeling approaches. Elevated lactate levels primarily reflect impaired tissue perfusion and metabolic stress, factors that are closely linked to rapid clinical deterioration. Consequently, lactate appears to be particularly informative for identifying patients at risk of early death rather than for predicting mortality as a binary outcome. This temporal sensitivity is more effectively captured by time-to-event analysis than by logistic regression [28].

Similarly, serum albumin and cortisol levels were not retained as independent predictors in the final multivariable logistic regression model but emerged as significant determinants of mortality timing in Cox analyses. Lower albumin levels may reflect a combination of malnutrition, systemic inflammation, and capillary leakage, all of which can accelerate adverse outcomes. Elevated cortisol levels likely represent an intensified stress response, which may be more relevant to the speed of clinical decline than to mortality occurrence alone. These findings suggest that nutritional and endocrine factors play a greater role in shaping the trajectory of sepsis rather than simply determining survival status [13,29].

The robust positive association between elevated baseline cortisol levels and an increased hazard of early mortality observed in our cohort holds significant implications for the ongoing clinical debate surrounding moderate-dose corticosteroid therapy in sepsis. In international guidelines, such as the Surviving Sepsis Campaign, the administration of intravenous hydrocortisone (typically at a moderate dose of 200 mg/day) is recommended only for patients with septic shock who remain hemodynamically unstable despite adequate fluid resuscitation and vasopressor therapy. The physiological rationale behind this targeted therapy is to overcome critical illness-related corticosteroid insufficiency (CIRCI) or systemic glucocorticoid receptor resistance. This approach is guided by major randomized controlled trials, which demonstrated that while moderate-dose hydrocortisone accelerates shock reversal and increases vasopressor-free days, its impact on ultimate all-cause binary mortality reduction varies depending on baseline patient case-mix and the severity of systemic shock [30,31]. Our findings demonstrate that patients with accelerated mortality trajectories already possess massive endogenous hypercortisolemia at baseline, likely representing an extreme, uncompensated counter-regulatory stress response. This supports the clinical paradigm that unguided, universal administration of exogenous steroids across all sepsis severities may be redundant or potentially detrimental due to the risks of prolonged immunosuppression and secondary metabolic derangements. Instead, moderate-dose corticosteroid interventions should remain strictly reserved for refractory shock states where tissue-level corticosteroid activity is functionally exhausted, emphasizing the potential future utility of tracking baseline endogenous steroid profiles to guide personalized critical care therapeutics.

Importantly, our findings should be interpreted within the context of the existing sepsis prognostic literature. The independent associations observed for SOFA score, PLT, lactate, albumin, and renal dysfunction are largely consistent with well-established predictors of adverse outcomes and therefore primarily validate the clinical characteristics of our cohort rather than represent novel observations. The principal contribution of the present study lies in its evaluation of adrenal hormonal biomarkers. Although baseline cortisol and DHEA demonstrated significant associations in univariable analyses and cortisol remained independently associated with time to death in the Cox model, the primary endocrine hypothesis was only partially supported because the cortisol/DHEA ratio did not retain independent prognostic significance after adjustment for established clinical predictors. These findings suggest that hormonal biomarkers may provide complementary pathophysiological and temporal information but should currently be regarded as adjunctive rather than replacement prognostic markers in sepsis.

Several limitations should be acknowledged. First, this was a retrospective, single-center study with a relatively limited sample size, which may restrict the generalizability of the findings. Second, although internal validation was performed using 1000 bootstrap resamples, no external validation cohort was available; therefore, the prognostic performance of the models should be considered internally validated but not externally confirmed. Third, the use of univariable screening followed by stepwise backward selection may increase the risk of model instability and overfitting, even though optimism-corrected performance estimates suggested only limited optimism. Fourth, missing data in some covariates required complete-case analysis for multivariable modeling, which may have introduced selection bias. Finally, because cortisol and DHEA measurements were obtained retrospectively from routine clinical data, dynamic endocrine responses over longer follow-up periods could not be fully evaluated. Future prospective, multicenter studies with external validation are needed to confirm these findings and clarify the clinical utility of endocrine biomarkers in sepsis prognosis.

## 5. Conclusions

In our study, baseline cortisol and DHEA levels were significantly higher among non-survivors, whereas the cortisol/DHEA ratio did not differ significantly between survival groups. Higher baseline DHEA was associated with time to death in univariable analysis. The cortisol/DHEA ratio does not provide an additional independent prognostic value over established clinical and laboratory parameters (e.g., SOFA score). These results demonstrate that the dynamics of individual biomarkers and organ failure scores remain the most decisive elements in evaluating the adrenal endocrine response in sepsis. However, given the exploratory, retrospective, single-center nature of the study and the absence of external validation, these results must be interpreted with caution. While our data highlight distinct acute changes in individual baseline adrenal steroid concentrations during severe systemic infection, established clinical indices of multi-organ failure and routine laboratory measures remain the most definitive elements for clinical risk stratification.

## Figures and Tables

**Table 1:** Baseline clinical, laboratory, and endocrine characteristics stratified by survival status.

Variable	Survivors (*n* = 32)	Non-Survivors (*n* = 74)	Missing, *n*	*p*
Demographic and clinical variables				
Age, years	70.31 ± 13.21	75.07 ± 13.74	0	0.098
Female sex, *n* (%)	19 (59.4)	36 (48.6)	0	0.398
SOFA score	7.97 ± 2.68	10.26 ± 2.48	0	<0.001
APACHE II score	24.06 ± 8.31	27.74 ± 9.74	0	0.051
GCS	9.72 ± 4.24	8.15 ± 4.09	0	0.082
Infection characteristics				
Infection source			0	0.786
Pneumonia	19 (59.4)	44 (59.5)		
Bloodstream infection	4 (12.5)	15 (20.3)		
Urinary tract infection	5 (15.6)	10 (13.5)		
Intra-abdominal infection	3 (9.4)	4 (5.4)		
Central nervous system infection	1 (3.1)	1 (1.4)		
Infection acquisition			0	0.091
Community-acquired	18 (56.2)	28 (37.8)		
Nosocomial	14 (43.8)	46 (62.2)		
Relevant treatments, *n* (%)				
Antibiotic therapy	32 (100.0)	74 (100.0)	0	1.000
Combination antibiotic therapy (≥2 agents)	18 (56.3)	55 (74.3)	0	0.073
Three-agent antibiotic therapy	3 (9.4)	16 (21.6)	0	0.172
Baseline laboratory variables				
WBC1	16.00 ± 7.72	14.41 ± 8.98	0	0.356
NEU1	84.13 ± 9.73	82.88 ± 10.69	0	0.559
HB1	11.38 ± 2.81	10.09 ± 2.30	0	0.027
PLT1	272.19 ± 105.83	210.41 ± 132.18	0	0.013
Urea1	83.47 ± 46.28	122.64 ± 89.32	0	0.004
Creatinine1	1.40 ± 1.21	1.50 ± 1.10	0	0.683
eGFR1	79.17 ± 64.40	88.67 ± 124.49	0	0.607
CRP1	149.14 ± 119.34	148.29 ± 94.84	2	0.972
PCT1	5.13 ± 9.74	8.89 ± 27.05	3	0.303
Lactate1	2.07 ± 2.40	3.61 ± 4.28	0	0.020
Bilirubin1	0.76 ± 0.75	0.90 ± 1.22	0	0.453
Albumin	3.03 ± 0.55	2.76 ± 0.77	0	0.047
Endocrine biomarkers				
Cortisol1	23.51 ± 13.77	36.94 ± 41.83	5	0.018
DHEA1	10.08 ± 8.57	15.67 ± 18.19	4	0.037
Cortisol/DHEA ratio	3.45 ± 2.46	3.91 ± 4.35	9	0.504
Cortisol2	19.76 ± 10.95	20.05 ± 9.59	52	0.927
DHEA2	9.38 ± 9.62	14.32 ± 16.12	48	0.154

**Table 2:** Multivariable logistic regression analysis for mortality.

Variable	β Coefficient	95% CI	Std. Error	OR	*p*
Intercept	−2.135	0.013–1.044	1.111	0.12	0.055
SOFA score	0.330	1.15–1.69	0.099	1.39	<0.001
PLT1	−0.0043	0.919–0.998	0.0021	0.996	0.042
Urea1	0.0106	1.020–1.212	0.0044	1.01	0.016

AUC: 0.7956, Null Deviance: 129.84, Residual Deviance: 103.05, Akaike Information Criterion (AIC): 111.05.

**Table 3:** Final multivariable Cox proportional hazards model for time to death after sepsis onset.

Variable	HR	95% Confidence Interval	*p* Value
SOFA score	1.14	1.03–1.27	0.014
PLT1	0.998	0.996–1.000	0.033
Cortisol1	1.014	1.007–1.022	<0.001
Lactate1	1.10	1.03–1.18	0.004
Albumin1	0.64	0.46–0.90	0.009

Model characteristics: *n* = 101 (5 NAs removed); deaths = 71; C-index = 0.744. Model fit: LR *p* = 3 × 10^−9^; Wald *p* = 10^−9^; Score *p* = 2 × 10^−11^. HRs greater than 1 indicate an increased hazard of death, whereas values below 1 indicate a protective effect. Variables were selected based on clinical relevance and stepwise Cox regression analyses.

## Data Availability

Data is contained within the article or Supplementary Materials. The original contributions presented in this study are included in the article/Supplementary Materials. Further inquiries can be directed to the corresponding author.

## Supplementary Materials

The following supporting information can be downloaded at: https://www.mdpi.com/article/10.3390/jcm15155969/s1, File S1: De-identified clinical characteristics, disease severity scores, and laboratory parameters of the sepsis study population.

## Abbreviations

The following abbreviations are used in this manuscript:

ACTH	Adrenocorticotropic hormone
AIC	Akaike Information Criterion
APACHE II	Acute Physiology and Chronic Health Evaluation II
AUC	Area under the curve
C-index	Concordance index
CI	Confidence interval
CRP	C-reactive protein
DHEA	Dehydroepiandrosterone
DHEAS	Dehydroepiandrosterone sulfate
eGFR	Estimated glomerular filtration rate
GCS	Glasgow Coma Scale
HB	Hemoglobin
HPA	Hypothalamic–pituitary–adrenal
HR	Hazard ratio
ICU	Intensive care unit
LOESS	Locally estimated scatterplot smoothing
LR	Likelihood ratio
NEU	Neutrophil percentage
NEWS	National Early Warning Score
OR	Odds ratio
PCT	Procalcitonin
PLT	Platelet count
ROC	Receiver operating characteristic
SIRS	Systemic inflammatory response syndrome
SOFA	Sequential organ failure assessment
VIF	Variance inflation factor
WBC	White blood cell count
